# A Case Report of Hemolytic Hyponatremia

**DOI:** 10.7759/cureus.53417

**Published:** 2024-02-01

**Authors:** Santiago Manrique-Castaño, Luisa Rodríguez-Rosero, Raúl Vallejo-Serna

**Affiliations:** 1 Internal Medicine, Hospital Universitario San Jorge, Pereira, COL; 2 Internal Medicine, Hospital Universitario del Valle "Evaristo García" - Universidad del Valle, Cali, COL

**Keywords:** sickel cell, sheehan’s syndrome, hypopituitarism, hereditary spherocytosis, anemia

## Abstract

Hereditary spherocytosis/elliptocytosis is a non-immune hemolytic anemia caused by an alteration in the erythrocyte membrane that predisposes the cell to its lysis. This report presents a case of a 42-year-old woman with a history of spontaneous abortion, associated with postpartum bleeding, chronic anemia, and premature menopause. After five years, she consulted due to alterations in the state of consciousness and severe symptomatic hyponatremia, with a diagnosis of hypopituitarism, explained by a late Sheehan syndrome. During hospitalization, she developed non-immune hemolytic anemia associated with a positive osmotic fragility test. A diagnosis of hereditary spherocytosis/elliptocytosis was made. We correlate blood hypoosmolarity as a trigger with the in vitro hypotonic solution of the osmotic fragility test for the diagnosis of this disease. This association is not reported in the literature; in our case, we show the concomitant improvement of anemia with the increase in sodium levels and hormonal replacement.

## Introduction

Hemolysis refers to the destruction of the erythrocytes before 120 days, which corresponds to their usual life cycle; it is called hemolytic anemia when hemolysis surpasses the erythropoiesis capacity. This type of anemia is classified by its mechanism: extravascular, which is mediated by the reticuloendothelial system, and intravascular, which involves direct cell destruction, fragmentation, and oxidation [1,2].

Non-immune hemolytic anemias include microangiopathic hemolytic anemia, anemia induced by infections or drugs, and anemia resulting from intrinsic defects of the red blood cell, the latter including spherocytosis and hereditary elliptocytosis, which are characterized by a defective erythrocyte membrane that leads to reduced deformability being selectively destroyed in the spleen [3]. Its diagnosis is typically made with the osmotic fragility test, wherein erythrocytes are exposed to hypotonic solutions in order to evaluate the degree of hemolysis [2,3].

The interaction of plasma osmolality and hemolysis resulting from defects in the erythrocyte membrane has not been clearly described. However, sodium being the extracellular cation of greatest importance for blood osmolality, it has been suggested that the low osmolality secondary to a decrease in serum sodium could trigger hemolysis in patients with spherocytosis [4]. Hyponatremia has several etiologies including hormonal disorders in which the dysfunction of the hypothalamic-pituitary axis is one of the main ones [4].

We present a case of a patient with severe symptomatic hyponatremia in the context of Sheehan syndrome, in whom non-immune hemolytic anemia secondary to hereditary spherocytosis/elliptocytosis was documented, considering the decreased serum osmolarity as a trigger for the anemia crisis, given the improvement in hemoglobin levels as osmolarity corrects.

## Case presentation

A 42-year-old Afro-descendant woman from a remote rural area of southwestern Colombia presented to our emergency department with drowsiness and multiple emetic episodes over the last week. A thorough physical examination showed altered consciousness (drowsiness), dehydration and jaundice, stiff skin, and hair loss in the outer third of the eyebrow. The patient’s temperature was 37.1°C, blood pressure 114/60 mmHg, heart rate 89 bpm, oxygen saturation 97%, and glucose level 65 mg/dL. Treatment with intravenous fluids, including a dextrose solution, was started.

The only known medical history included a spontaneous abortion in the second trimester of pregnancy five years earlier. Since then, she has been hospitalized three times for anemia, which necessitated red blood cell transfusions; however, no additional clinical history or studies for anemia were provided. Additionally, the patient experienced premature menopause at 38 years of age and a decreased libido, with no other relevant medical history.

**Figure 1:**
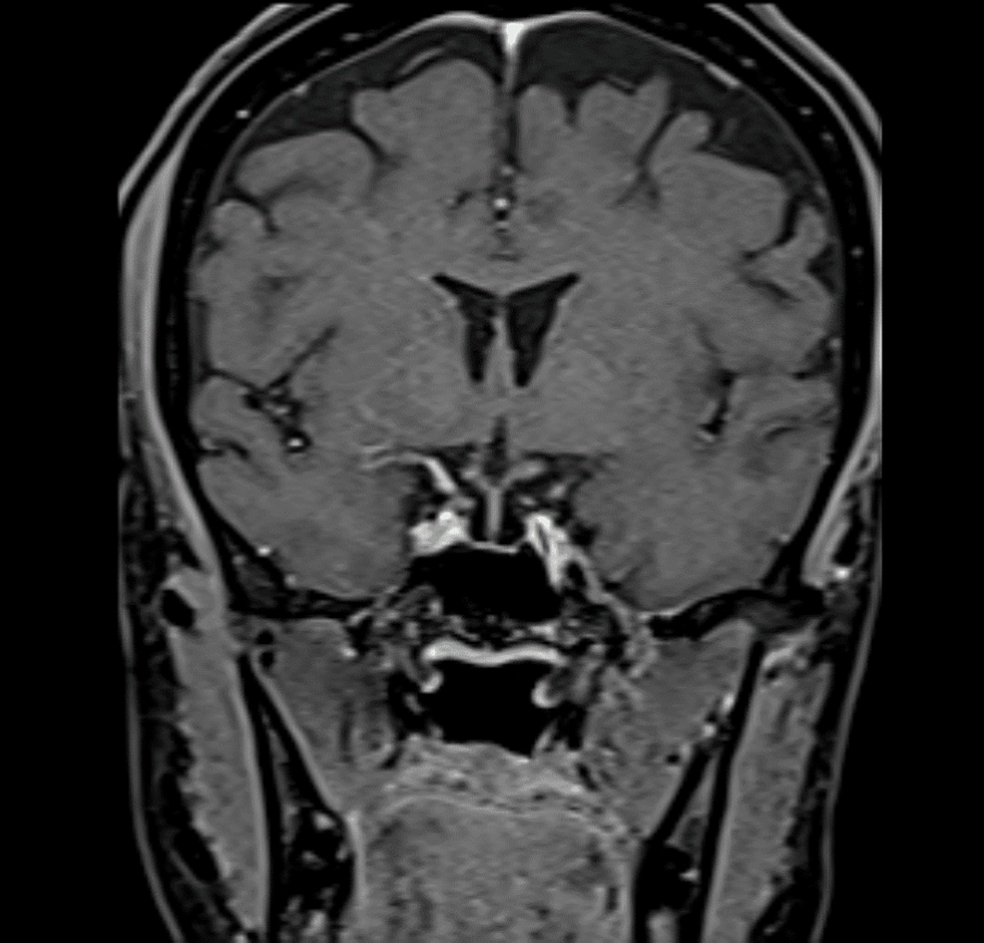
Patient's MRI of the sella turcica T1 coronal MRI of the patient with the finding of an empty sella turcica.

Laboratory tests on admission showed anemia with hemoglobin of 6.5 g/dL, a mean corpuscular volume of 83 fL, a mean corpuscular hemoglobin of 29 pg, hypochloremia, and severe hyponatremia (Table 1). Hyponatremia was initially interpreted as secondary to emesis; however, after achieving an adequate hydration state, it persisted. Studies for etiology were expanded, including hormonal profile, which showed panhypopituitarism (Table 1). Given these findings, brain magnetic resonance imaging was performed with the finding of an empty sella turcica supporting the diagnosis (Figure 1). Therefore, in the context of the history of abortion and panhypopituitarism, the diagnosis of Sheehan syndrome was made, which explained the euvolemic hyponatremia [5]. A hormonal replacement therapy was started with levothyroxine and prednisone.

**Table 1:** Laboratory tests of the patient The values ​​in parentheses correspond to the upper and lower limits for each result.

Laboratory tests			
Leukocytes	4500 (3980-10040)	Thyroid-stimulating hormone	0.37 UI/mL (0.4-4.05)
Neutrophils	2600 (1560-6130)	Free thyroxine (T4)	<0.07 ng/dL (0.78-2.19)
Lymphocytes	1700 (1180-3740)	Cortisol AM	4.1 mcg/dL (4.46-22.7)
Hemoglobin	6.5 g/dL (11.2-15.7)	Luteinizing hormone	1.7 mU/mL (13.1-86.5)
Hematocrit	18.9% (34.1-44.9)	Follicle-stimulating hormone	3.8 mU/mL (21.5-131)
Mean corpuscular volume	83.9 fL (79.4-94.8)	Growth hormone	<0.05 ng/mL (0.06-6.8)
Mean corpuscular hemoglobin	29 pg (25.6-32.3)	Prolactin	5.4 ng/mL (1.8-20.3)
Platelets	211.000 (182000-369000)	Sodium	114 mmol/L (137-145)
Total bilirubin/direct	3.4 mg/dL/0 mg/dL	Chloride	78 mmol/L (98-107)
Lactate dehydrogenase	618 U/L (120-246)	Potassium	3.6 mmol/L (3.5-5.1)
Haptoglobin	<8 mg/dL (41-165)	Creatinine	0.5 mg/dL (0.66-1.25)
Reticulocyte Production Index	2.6%	Ureic nitrogen	2mg/dL (7-17)
Hemoglobin electrophoresis	Hemoglobin A: 98%, Hemoglobin A2: 2%		
				Peripheral blood smear	Red series, hypochromia, and moderate poikilocytosis: elliptocytes ++, keratocytes +, some dacryocytes, and acanthocytes		
White series, leukocyte count, and normal morphology							
			Platelets, normal count, and morphology		

**Figure 2:**
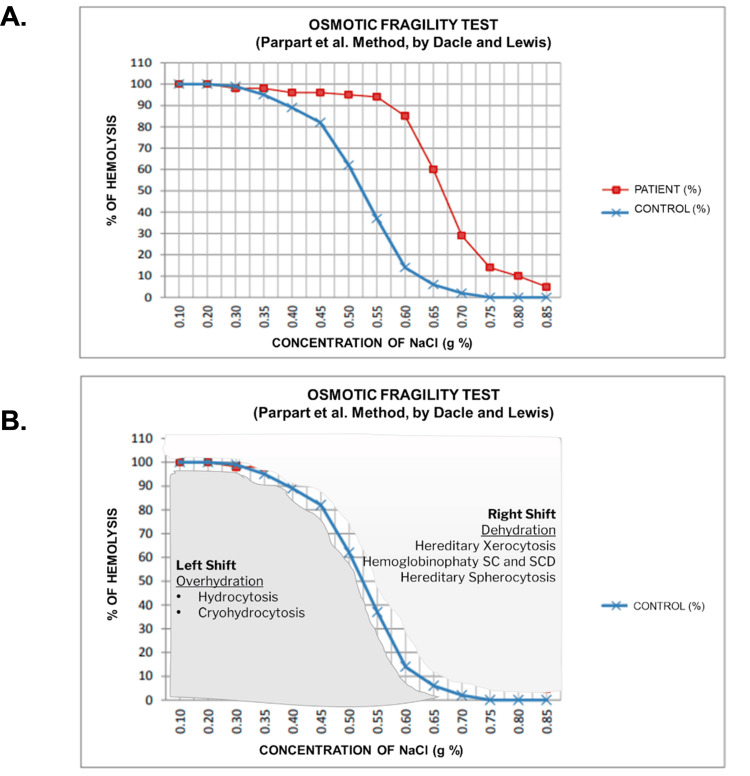
Osmotic fragility test (A) Patient's osmotic fragility test. Blue represents the control of the test in a healthy patient. Red corresponds to the patient's test, where the curve is shifted to the right, meaning that with lower NaCl concentration there is a higher percentage of hemolysis compared to the control. (B) States of dehydration and overhydration of the erythrocyte [8]. NaCl: sodium chloride; SCD: Sickle cell anemia

Regarding anemia, the studies suggested non-immune hemolytic anemia (Table 1), with a peripheral blood smear showing poikilocytosis and elliptocytes. The direct Coombs test was negative on two occasions; thus, non-immune hemolytic anemia was contemplated for which studies were started, ruling out hemoglobinopathies and paroxysmal nocturnal hemoglobinuria. Bone marrow studies were normal. Chronic infections (HIV, syphilis, hepatitis B and C, HTLV I-II) as well as systemic autoimmune disease were ruled out. An osmotic fragility test was performed, which showed early hemolysis with a slightly hypotonic solution (Figure 2A); it was concluded positive for a hereditary membranopathy spherocytosis/elliptocytosis type, but genetic studies were not possible.

Decreased serum osmolarity (calculated at 232 mOsm/L) was regarded as a triggering factor for the hemolysis episode, which showed progressive improvement with hormone replacement that simultaneously improved the hemolysis profile and hemoglobin levels. The patient was discharged with hormone replacement and folic acid supplementation, with hematology follow-up indication; however, the evolution is unknown.

## Discussion

We present a clinical case that leaves us with great lessons. First, through the history and detailed approach of hyponatremia, the diagnosis of late Sheehan syndrome was achieved. Second, it must be recalled that anemia is a secondary condition, and a prudent and timely approach leads us to the etiology, as in this case a hereditary membranopathy spherocytosis/elliptocytosis type in an adult [2,5].

In Sheehan syndrome, there is necrosis of the pituitary cells that occurs secondary to hypovolemic shock in postpartum hemorrhage [5], and it also has been described as secondary to late abortions. During pregnancy there is an increase in the gland volume mainly due to the hyperplasia of lactotroph cells, which enlarge by 45% in the first trimester and from 120% to 136% towards the end of pregnancy, increasing the need for glandular blood flow. In scenarios of severe hypotension, it is impossible to satisfy blood flow due to increased demands, generating infarction of the gland. This has been classically described in the pathophysiology of early Sheehan syndrome [5]. There is a complementary theory: it is the sequestration of antigens in the pituitary gland after an initial injury such as postpartum hemorrhage, which over time generates destruction and, as a consequence, panhypopituitarism. The latter could explain the clinical presentation of our patient five years after an abortion [5,6].

This syndrome is described as panhypopituitarism, evidenced in our case with a deficiency of luteinizing hormone, follicle-stimulating hormone, thyroid-stimulating growth hormone, cortisol, and prolactin [5]. In magnetic resonance imaging, there was the most common finding, an empty sella turcica [5,6]. The diagnosis is sometimes delayed, often presenting as decreased libido, hair loss in the outer third of the eyebrow, altered consciousness, and loss of body hair. In laboratory tests, we can find altered hormonal euvolemic hyponatremia due to a syndrome of inappropriate antidiuretic hormone release, hypothyroidism, or hypocortisolism [4].

With regard to anemia in Sheehan syndrome, it can present in up to 80% of the cases, with 44% due to iron deficiency and the other percentage corresponding to a multifactorial type, among which hypothyroidism is included. However, there are no clear records of hemolytic anemia, which implicated the need to search for other types of etiologies [6].

There are different causes of hemolysis, those mediated by autoimmunity, where antibodies facilitate phagocytosis and destruction of cells by the complement system [1], and non-immune hemolytic anemias that include: microangiopathic hemolytic anemia, anemia induced by infections or drugs, and anemia resulting from intrinsic defects of the red blood cell, such as hemoglobinopathies, enzymopathies, and membranopathies, which are mostly inherited, with the exception of paroxysmal nocturnal hemoglobinuria [1,2].

In membranopathies, interactions between the cytoskeleton and the lipid bilayer are compromised due to a deficiency of proteins, such as ankyrin, α and β-spectrin, protein 4.2, and band 3, leading to membrane destabilization and release of microvesicles that cause a reduction in its surface area, its volume and, lastly, the lysis of the erythrocyte. The diagnosis is carried out through the osmotic fragility test, which consists of exposing the erythrocytes to solutions with a progressively decreasing tonicity as a means to evaluate the degree of hemolysis; in these conditions, the cells begin to hemolyze at lower concentrations of sodium chloride (NaCl) compared to healthy red blood cells. It has a diagnostic performance with a sensitivity of 66% and a specificity of 81% [7]. This test allows us to evaluate disorders of dehydration and overhydration of the erythrocyte that end in its lysis in a hypo or hypertonic environment, as evidenced in Figure 2 [8,9].

Hereditary spherocytosis with autosomal dominant inheritance can manifest at any age; however, it has been classically described in childhood. In adults, it is common to find a history of jaundice, anemia, cholelithiasis, splenomegaly, or a splenectomy. Deficiencies in the band 3 proteins correlate with late presentations. This has been explained by ectacytometer, demonstrating less deformity in erythrocytes than those with mutations in spectrin and ankyrin compared to band 3 [9].

**Figure 3:**
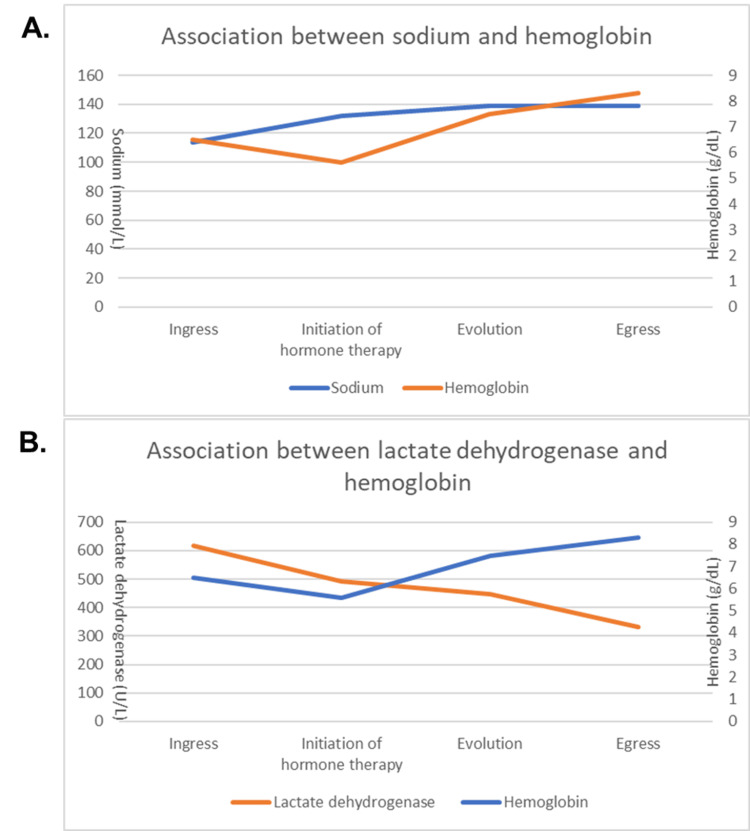
Evolution according to hormone replacement (A) Relationship between serum sodium and hemoglobin, showing an improvement of hemoglobin levels with sodium levels and the initiation of hormone therapy. (B) Relationship between lactate dehydrogenase and hemoglobin levels, showing improvement in hemolysis.

We describe a clinical case where we found a patient with a history of anemia, who in a state of severe hyponatremia and low serum osmolality presented a severe hemolytic crisis. From the physiological point of view, it is correlated with the in vitro osmotic fragility test [3]. We carried out the correlation between the hemoglobin and sodium levels and the lactate dehydrogenase and hemoglobin levels, as noted in Figure 3.

A search was carried out for the relationship between hereditary spherocytosis/elliptocytosis and hyponatremia or hypoosmolar states, but no results were found. However, it has been reported that worsening of hemolysis occurs in stress situations, such as low pH and low concentrations of glucose and adenosine triphosphate [8]. Spherocytosis/elliptocytosis is characterized by chronic dehydration of the erythrocyte; under normal conditions, it contains intracellular sodium at around 10 mEq/dL compared to the plasma which has an average of 140 mEq/dL. The regulation of intracellular potassium (K) and sodium (Na) is maintained by the Na-K ATPase pump. The ion permeability of the erythrocyte membrane is very low, which prevents alterations in cell volume driven by osmotic changes. In hereditary spherocytosis/elliptocytosis, the membrane is altered, leading to a passive leak of cations and the release of water; as a consequence, these erythrocytes maintain a reduced cell volume with chronic dehydration. Exposure to a hypotonic environment favors the entry of water into the diseased erythrocyte and its rupture due to having less tolerance to volume changes [3,8]. We thus describe this clinical correlation between sodium levels and hemolytic crisis in our patient [3,8].

## Conclusions

Finally, we refer to the diagnosis as hereditary spherocytosis/elliptocytosis since the presentation in the peripheral blood smear was characterized by the presence of elliptocytes, and the osmotic fragility test was positive in both entities. Genetic studies were requested to determine the type of membranophathy; however, they were not conducted by the patient. The treatment for both entities is similar, involving folic acid supplementation and, in severe cases, splenectomy.
